# Bone quality and growth characteristics of growth plates following limb transplantation between animals of different ages - Results of an experimental study in male syngeneic rats

**DOI:** 10.1186/1749-799X-6-53

**Published:** 2011-10-14

**Authors:** Hitesh N Modi, Seung Woo Suh, Boopalan Prjvc, Jae-Young Hong, Jae-Hyuk Yang, Young-Hwan Park, Jae-Moon Lee, Yong-Hyon Kwon

**Affiliations:** 1 Scoliosis Research Institute, Department of Orthopedics, Korea University Guro Hospital, Seoul, Korea

**Keywords:** Limb transplant, isochronografts, heterochronografts, osteoporosis, growth potentials of growth plate

## Abstract

**Introduction:**

The purpose of this study was to identify graft osteoporosis post transplantation by micro-CT analysis, and the growth potential of growth plates in the transplanted limb.

**Methods:**

Ten juvenile to juvenile and five juvenile to adult hind limb transplants were performed in male syngeneic Lewis rats. Upper tibial bone density in isochronograft and heterochronograft limbs was measured by 3D micro-CT and compared with that of the opposite non-operated limbs.

**Results:**

We observed inferior bone quality (p < 0.05) in heterochronografts compared to isochronografts. After transplantation, isochronografts did not exhibit increases in tibial lengths compared to opposite juvenile non-operated tibias (p = 0.66) or heterochronograft tibias (p = 0.61). However, significant differences were observed between heterochrongraft tibial lengths when and opposite adult non operated tibial lengths (p < 0.001).

**Conclusions:**

Age dependent alterations affect bone quality, resulting in post transplantation osteoporosis in heterochronografts, but not isochronografts. However, the growth plates of transplanted limbs retain their properties of longitudinal growth and continue to grow at the same rate.

## Background

Osteoporosis often develops in transplanted and grafted bone after bone transplantation or grafting [1-4] due to revascularization and creeping substitution that occur during the repair process. Kline et al. [1] proposed that lack of weight bearing post transplantation and subsequent stress shielding are the causative factors of post transplantation osteoporosis.

Increases in the length of long bones occur by enchondral ossification at the growth plates (metaphyses). Each growth plate has an inherent mechanism for determining growth rate and limb morphology [1]. In addition, growth at the physes is influenced by a variety of hormones that have permissive effects and enable the growth plated to achieve their maximum growth potential. C growth hormone, thyroxine, somatomedin C (insulin like growth factor I), insulin like growth factor II, cortisol, insulin, and sex hormones all play important roles in regulating growth plate development and rates of limb growth [5-9]. There is a complex interaction between these different hormonal factors. Levels of these hormones within the body show remarkable differences during growth phases (immature skeleton) and adult phases (completed skeletal maturity). But what happens when a growth plate with significant growth potential is placed in an adult hormonal environment? Will it retain its property of promoting longitudinal growth in the changed hormonal milieu? If yes, then is this increase in length different from the increase in length occurring in a juvenile hormonal environment?

Previous studies have attempted to understand the behavior of juvenile growth plates in adult bodies by limb transplants between animals of different ages. However, they yield conflicting results; some report that juvenile growth plates attain full growth potential in the adult body[1,10] while others report that juvenile growth plates attain only partial growth potential in the adult hormonal environment[11,12]. Vascularized growth plate transplants are now feasible alternatives for the management of growth plate damage by tumor, infection, trauma or congenital anomalies. Previous reports indicate that microvascular epiphyseal transplants may successfully be used to reconstruct the extremities of children whose epiphyseal plates were damaged or surgically removed as a result of disease or trauma [13-17]. These studies demonstrated both the reconstruction of bony defects and restoration of longitudinal growth.

The performance of limb transplants between male syngeneic Lewis rats of different ages constitutes a unique experimental model, and is made possible by advances in microvascular surgery that allow us to study the pathogenesis of post bone transplantation osteoporosis. Therefore, the purpose of this study was, to determine the level of osteoporosis using micro CT post limb transplantation in this animal model, and to determine the effects of age on growth plate potential.

## Methods

After obtaining approval from the University Animal Care and Use Committee (ACUC No BU08026 we obtained male syngeneic Lewis rats for use in the present study. Two groups of rats that differed only in age were used in the present study. The first group consisted of 10 recipient juvenile rats (mean age 23.8 days, range 21-28 days). The second group consisted of 5 recipient adult rats (mean age 72.4 days, range 71-77 days). The hind limbs of these rats were divided into two experimental groups as follows:

Group 1: Microvascular transplants of the right hind limbs of 10 juvenile donors into 10 juvenile recipients served as isochronografts.

Group 2: Microvascular transplants of the right hind limbs of 5 juvenile donors into 5 adult recipients served as heterochronografts.

All surgical procedures were performed under general anesthesia, which was induced and maintained by administering single injected intraperitoneal doses of ketamine. Two surgical teams were involved: one team was dedicated to harvesting the donor limbs from 15 juvenile rats while the other performed microvascular anastomosis of the donor limbs to the recipient stumps of 10 juvenile and 5 adult rats. The donor juvenile limbs were harvested by above knee amputation at the mid-femur diaphysis level. The femoral vessels of the donor limbs were the last structures divided in an attempt to limit the ischemic time to less than 1 hour. This was followed by microvascular anastomosis of the donor limb to the recipient above knee (AK) amputation stumps of 10 juvenile and 5 adult rats. Bony stabilization was first achieved by inserting an intramedullary 18G needle into the femur. This was followed by microvascular anastomosis of the femoral arteries with 10-0 nylon interrupted sutures. Femoral and sciatic nerve anastomosis were then performed with 10-0 nylon, and finally the corresponding muscle groups were sutured. All rats received prophylactic antibiotics in the form of intramuscular injections of penicillin, one dose postoperatively per animal.

**Figure 1:**
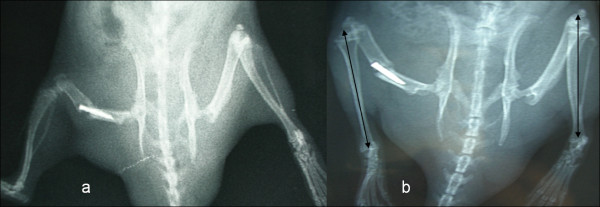
**Radiogram of rat limbs after 3 and 10 weeks of transplantation**. **a**: Radiogram of rat hind limbs taken 3 weeks after transplantation - tibial lengths of both transplanted and opposite non operated limbs were measured. **b**: Radiograph of rat hind limbs 10 weeks after transplantation, showing the increased length of the tibias of transplanted limbs as well as opposite non operated limbs.

Post transplantation, the rats were housed separately and were allowed to bear weight on the transplanted limbs as pain allowed. Food was dispensed above a 15° inclined platform so that the animals would be forced to bear weight to reach the food. All operated animals were observed engaging in this behavior. Increases in the lengths of the transplanted limbs were then monitored by calculating tibial lengths using serial postoperative anteroposterior radiograms. Tibial length was measured from the joint lines at the knee and ankle joints, which included the highest growth potential areas of the epiphyses. While taking radiograms, the rats were sedated with single doses of intraperitoneal ketamine and placed in the prone position with both knee joints in flexion, with the anterior side of the tibia facing the X-ray tube. The first set of radiograms was taken 3 weeks after transplantation (Figure 1a). All animals were sacrificed 10 weeks post transplantation by injection overdose of intraperitoneal thiopentone. The second and last set of radiograms was taken at this time (Figure 1b). The increases in tibial length of the transplanted limbs were measured by calculating the difference between the lengths of the tibia at the 3- and 10-week post-transplantation radiograms. Similarly, the increases in tibial length of the contralateral non-operated hind limbs during the same time interval were also calculated. In order to ensure accuracy, all radiographs were scanned and tibial lengths were measured using the software program Rapidia Version 2.7 (INFINITT, Seoul, Korea).

The following results were evaluated separately:

**Sub group 1 **- Comparisons of the increases in tibial lengths of transplanted isochronograft (juvenile to juvenile) limbs, and increases in tibial lengths of the corresponding contralateral non-operated hind limbs in the same animals.

**Sub group 2 **- Comparisons of the increases in tibial lengths of transplanted isochronograft (juvenile to juvenile) limbs and transplanted heterochronograft (juvenile to adult) limbs.

**Sub group 3 **- Comparisons of the increases in tibial lengths of heterochronograft limbs and corresponding contralateral non-operated hind limbs in adult animals.

Limbs from completely separate animals (e.g., juvenile to juvenile isochronografts versus juvenile to adult heterochronografts; sub group 2) were compared using Student's t-test for independent groups, while limbs of the same animal (e.g., juvenile to juvenile isochronografts versus contralateral nonoperated limbs of the juvenile animals; sub group 1) were compared using Student's *t *test for correlated groups. P-values less than 0.05 were considered significant.

**Figure 2:**
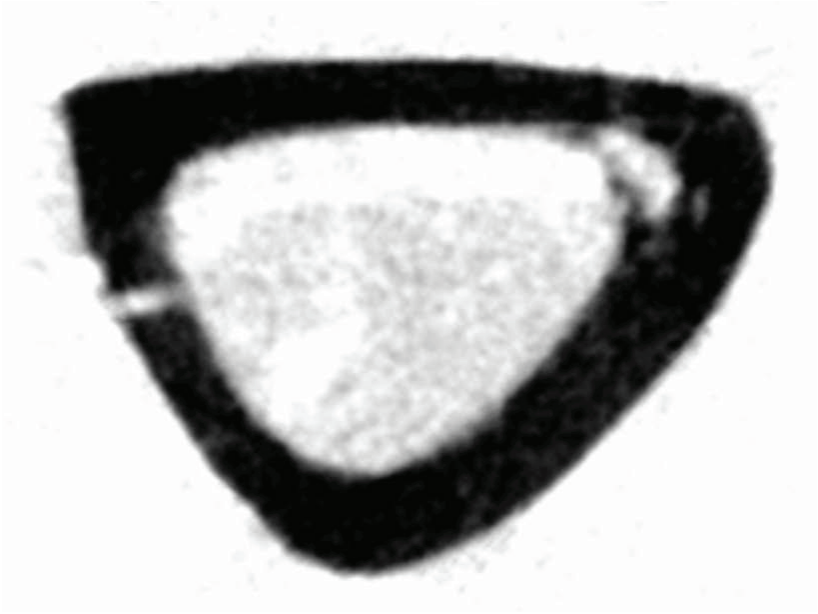
**3-D micro CT images of tibial diaphysis**. 3-D micro-CT image taken at the upper third of tibial diaphysis.

To assess the bone quality in the transplanted limbs, the tibiae of both isochronografts and heterochronografts were harvested from sacrificed animals. After stripping the harvested tibiae of all soft tissue the bones were prepared by precision sawing and subjected to a high resolution 3-dimensional micro-CT analysis (μ-CT-40, Scanco Medical AG, Zurich, Switzerland) at the upper third portion of the tibial diaphyses. The scanned images were used for 3-dimensional reconstruction of cubic voxel sizes 31 × 31 × 31 μm^3 ^(Figure 2). Each three dimensional image dataset consisted of approximately 200 micro-CT slide images (1024 × 1024 pixels) with 16 bit gray levels. Micro CT images were segmented using previously described methods [18]. Using accurate three dimensional data sets, bone volume fraction (VF), trabecular number (Tb.N), trabecular thickness (Tb.Th), trabecular separation (Tb.Sp), bone surface to total volume ratio (BS/TV) and bone surface to bone volume ratio (BS/BV) were calculated based on unbiased, assumption free, 3-dimensional methods. The results were presented as mean and SD. The micro CT parameter values in the isochronograft group were then compared with the heterograft group using unpaired Student's t-tests. P-values less than 0.05 were considered significant.

## Results

### The micro-CT 3D parameters of transplanted bone in isochronograft and heterochronograft limbs for measuring osteoporosis (Table 1)

**Table 1:** The micro-CT 3D parameters of transplanted bone in isochronograft and heterochronograft limbs.

	Isochronograft(n = 10) Mean ± SD (Range)	Heterochronograft(n = 5) Mean ± SD (Range)	*t *value	p value
**BV/TV**	0.3 ± 0.177 (0.09-0.38)	0.055 ± 0.023 (0.028-0.081)	3.03	0.0096
**Tb.N (1/mm)**	3.924 ± 1.249 (1.563-5.650)	1.42 ± 0.56 (0.819-1.862)	4.21	0.0010
**Tb.Th (mm)**	0.0697 ± 0.0242 (0.037-0.112)	0.0421 ± 0.003 (0.039-0.047)	2.47	0.028
**Tb. Sp (mm)**	0.221 ± 0.15 (0.083-0.583)	0.757 ± 0.308 (0.421-1.174)	-4.63	0.0005
**BS/TV (1/mm)**	10.1 ± 2.22 (7.197-13.295)	2.69 ± 1.10 (1.419-3.863)	7.01	< 0.0001
**BS/BV (1/mm)**	35.4 ± 9.40 (22.70-53.35)	48.9 ± 1.76 (9.257 to 54.27)	-3.13	0.0080

**Abbreviations**: Tb.N = Trabecular number; Tb.Th = Trabecular thickness; Tb.Sp = Trabecular Separation; BV = Bone Volume; TV = Tissue Volume; BS = Bone Surface; ns = no significance, SD = Standard Deviation.

The parameter values of bone mass indicated significantly inferior bone quality of transplanted bone in the heterochronograft group as compared to the bone quality in the isochronograft group. The 3D fractional bone volume (VF) in the heterochronograft group was significantly less than the bone volume fraction of the isochronograft group (p = 0.009). In the heterochronograft group, the values of Tb.N were significantly less (p = 0.001) than the values in the isochronograft group. The values of Tb.Sp were significantly increased in the heterochronograft group compared to the isochronograft group (p < 0.001) indicating less trabecular density in the heterochronograft group than the isochronograft group. The parameters of Tb.Th were significantly less in the heterochronograft group compared to the isochronograft group (p = 0.028) indicating thinner and weaker trabeculae in the heterochronograft group than the isochronograft group. The BS/TV in the heterochronograft group was significantly smaller than that of the isochronograft group (p < 0.001). Finally the BS/BV was significantly higher (p = 0.008) in the heterochronograft group compared to the isochronograft group.

### Comparisons of increases in length of transplanted limbs among the different subgroups (Table 2)

**Table 2:** Data show animal groups and increases in tibial lengths during the experimental period.

	n	Starting length (cm)	Final Length (cm)	Average net growth (cm)
**Juvenile isochronografts**	10	3.52 ± 0.24 (3.3-3.8 cm)	4.75 ± 0.21 (4.4-5.2 cm)	1.23 ± 0.13 (1.0-1.4 cm)
**Juvenile contra lateral limbs**	10	3.58 ± 0.32 (3.5-3.9 cm)	4.86 ± 0.23 (4.8-5.6 cm)	1.28 ± 0.19 (1.0-1.7 cm)
**Juvenile-adult heterochronografts**	5	3.56 ± 0.36 (3.4-3.9 cm)	4.78 ± 0.61 (3.9-5.3 cm)	1.22 ± 0.18 (1.0-1.4 cm)
**Adult contra lateral limbs**	5	4.58 ± 0.43 (4.1-5.1 cm)	5.08 ± 0.38 (4.8-5.7 cm)	0.50 ± 0.30 (0.0-0.80 cm)

There were similar increases in the lengths of juvenile isochronograft limbs and non-operated contra lateral hind limbs (sub group 1). During the experimental period the tibiae in the juvenile isochronograft limbs grew in length by 1.23 ± 0.13 cm (range, 1.0-1.4 cm). This was not significantly different (p = 0.66) (table 3) from the increases in tibial length of the non-operated contralateral hind limbs of these juvenile animals, which grew by 1.28 ± 0.19 cm (range, 1.0-1.7 cm) during the same period. Similarly, there was an increase in the length of the juvenile to juvenile isochronograft hindlimbs when compared to the increase in length of the juvenile to adult heterochronograft hind limbs (sub group 2). The tibiae of the juvenile isochronograft hind limbs grew in length by 1.23 ± 0.13 cm (range, 1.0-1.4 cm) during the experimental period. This was not significantly different (p = 0.61) (table 3) from the increases in tibial length of the juvenile to adult heterochronograft hindlimbs, which grew by 1.22 ± 0.18 cm (range, 1.0-1.4 cm) during the same time interval. However, the increases in length of juvenile to adult heterochronograft hindlimbs, were different compared to the increases in length of adult non-operated contra lateral hind limbs (sub group 3). During the experimental period, the tibiae of the juvenile to adult heterochronograft hind limbs increased in length by 1.22 ± 0.18 cm (range, 1.0-1.4 cm) which was significantly different (p < 0.001) (table 3) from the increase in tibial length of the adult non-operated contra lateral hind limbs, which increased in length by 0.50 ± 0.30 cm (range, 0-0.8 cm) during the same time interval.

**Table 3:** Comparison between tibial length increases of various groups during the experimental period.

	*t *value	p value
**Juvenile isochronograft vs juvenile contra lateral non-operated limb (Sub Group 1)**	- 0.447	0.66
**Juvenile isochronograft vs juvenile-adult heterochronograft (Sub Group 2)**	0.529	0.61
**Juvenile-adult heterochronograft vs adult contra lateral non-operated limb (Sub Group 3)**	-5.6	<0.001

## Discussion

Through our experimental study we found that the decrease in bone quality typically following limb transplant depends in part on recipient factors. As measured by micro-CT, all parameters of bone quality were significantly better in limbs transplanted between juvenile rats than between limbs transplanted from juvenile rats to adult rats, indicating that age related alterations in the hormonal milieu in the adult rats resulted in inferior bone quality in the transplanted limbs. To our knowledge, measuring bone quality using 3-D micro-CT, and comparing bone growth simultaneously, was performed for the first time in this study, which makes this study unique.

Kline et al. [1] noted that osteoporosis occurred following limb transplantation between animals of different ages; however, that study primarily dealt with the growth characteristics of growth plates following limb transplantation, without establishing the relationship between the degree of osteoporosis and the age of the recipient animal. They attributed the post transplantation osteoporosis to factors relating to both the non-weight-bearing, and lack of external stress conditions following limb transplantation. However, their hypothesis may be incorrect, because in a previous hindlimb transplanted rat model there were no significant differences in functional improvement between rats that received physiotherapy and rats that did not, except for foot drop [19]. However, they did not evaluate bone quality in this study.

From the results of our experimental study we found that host dependent age related hormonal factors play a vital role in the etiopathogenesis of post transplantation osteoporosis. Gourian et al. [20] showed that age related changes in bone mineral content (BMC) are due to the mineralization process itself and not imbalance in the remodeling process. Tissue age can vary within the same bone specimen due to reabsorption of bone by osteoclasts and formation by osteoblasts. Juvenile bone placed in an adult hormonal environment (heterochronografts) suffers much greater bone loss than juvenile bone placed in a juvenile hormonal environment (isochronografts). Of course, other factors such as circulation, neuronal control, bodily responses to stress and transplantation also play roles in maintaining the bone quality of transplanted allogenic bone from the donor.

The results of the above experiment suggest that even after transplanting the limbs of juvenile animals into adult animals (heterochronografts), the growth plates in the transplanted limbs retained their properties of longitudinal growth and continued to grow at the same rate in the new adult environment as they would have in the juvenile environment. The increase in length of the heterochronograft limbs was not significantly different from the increase in length of the isochronograft limbs. In addition, the increase in length of the isochronograft limbs was not significantly different from the increase in length of the non-operated contralateral hind limbs. Our results are similar to those of Kline et al. [1], who reported that a mature hormonal environment does not inhibit the longitudinal growth of immature growth plates. Kline et al. observed maintenance of growth in juvenile limbs transplanted into adult rats. They also studied growth plate morphology in transplanted limbs, and observed that all transplanted limbs demonstrate maintenance of growth plate morphology and columnar organization [1]. However, they did not assess the bone quality among these groups.

The increase in length of the heterochronograft limbs was, however, significantly greater than the increase in length of the non-operated contralateral hind limbs of the adult rats. In other words, after the age of 3 weeks, the internal environment of the host ceases to have a decisive role in the determination of the growth characteristics of the growth plate, and the increase in length of the growth plate is primarily determined by local transplantable factors that are expressed prior to transplantation by interactions between the inducing factors and inherited genomes. We can explain the increase in length of the adult non-operated limbs by the fact that the growth pattern in rats differs from that in humans, in that the growth plates in rats remain open later into adult life, though the growth rate at 10 weeks of age is a fraction of the rate at 3 weeks of age [21,22]. The temporal analysis of rat growth plate shows cessation of growth with age, despite the presence of a physis [23]. For this reason, we should be cautious regarding blind extrapolation of these results to humans, and rather, emphasize that these findings need to be confirmed in a clinical setting. Chiu et al. [12], in a similar study involving limb transplantation between animals of different ages, observed that the transplanted bone achieved only 70% of the normal growth in length. This finding was corroborated by Drzewiecki et al. [11] who also found that after limb transplantation the transplanted bone could not achieve normal growth potential, but noted that the maximum growth (91% of normal) was observed in heterochronografts. However, in both of these studies the nerves of the transplanted limbs were not sutured, and the animals were non-weight bearing, so the failure to achieve full growth could be attributed to the effects of denervation and lack of external stress. However, in our experiment blood vessels and nerves were sutured with microvascular anastomosis, minimizing the ischemic time, and therefore we could allow weight bearing in the subject group.

Stevens et al. [24] studied the growth of epiphyseal plate allografts after microvascular transplantation in rabbits of different ages, and found that the growth rate depended on the age of the donor epiphyseal plate and was independent of the age of the recipient. Glickman et al. [25] studied epiphyseal growth after microvascular transplantation to sites of different growth potential, and reaffirmed that growth potential of an epiphyseal plate transplant is a function of the donor, irrespective of the recipient site to which it is transplanted. Our report would further support their findings that the growth of epiphysis is an inherent property of the donor, while the quality of bone depends upon the internal environment of recipient's body.

There are reports in which microvascular epiphyseal transplants have been used to reconstruct the extremities of children whose epiphyseal plates were damaged or surgically removed as a result of disease or trauma. Vilkki [13] performed microvascular transplantations of the metatarsophalangeal joint with whole metatarsal bone in the treatment of radial club hand in nine children. At the average follow-up of six years they found that the deformity of the wrist had been reduced and growth of the ulna had been maintained due to intact functions of transplanted metatarsophalangeal joints. Innocenti et al [14-17] described the treatment of loss of the distal part of the radius, including the physis and epiphysis in skeletally immature patients, by performing vascularized proximal fibula transfers based on the anterior tibial artery. This included the physis, and a variable length of the diaphysis, and found a consistent and predictable longitudinal growth of the transferred fibula. On the basis of these findings, Innocenti et al. proposed that vascularized epiphyseal transfer is the only possible procedure that can solve the dual problem of replacement of osseous defect and restoration of longitudinal growth in the case of loss or damage to epiphyseal plates. Our report also supports the clinical implications of transferring intact joints with epiphyseal growth potentials on various congenital disorders, like epiphyseal dyplasia, tibial hemimelia, and psuedarthorosis of tibia (type IV), in which transplanted limbs could continue growth due to the intact inherent properties of epiphyseal growth plates from the donor, while bone quality would match the donor bone quality.

The major limitation of this study is that we did not use any control groups in our study, such as the comparison of bone and epiphyseal growth properties in a sham transplantation group. However, if we had used a control group with sham transplantation, all transplanted limbs would have necrosed due to lack of vacularity and lack of use. Another weak point is that we did not perform dynamic bone histometric parameters (after tetracycline labeling) of the healthy donor and recipient as well as of the transplanted limbs. We should have performed micro CT in the contralateral limbs of both groups to assess differences in limb bone quality. So far there are no clinical reports of allogenic transfers of growth plates. Additionally, the numbers of animals in recipient adult and juvenile groups were not equal and small, mainly due to limitations of funds. However, this is the first study in which micro CT was used to assess transplanted limb bone quality. Additionally we found consistent results in each experiment. Therefore we suggest further research on this issue, taking into consideration all of these weak points, to confirm our results. However, this study will provide useful information should such a procedure become feasible in the future.

## Conclusions

Although inherent recipient properties such as circulation, neuronal control, bodily responses to stress and transplantation play important roles in the fates of transplanted limbs or epiphyseal plates, bone quality following bone transplants also depends on recipient age; however, longitudinal growth remains unaffected by the recipient's age and continues in accordance with its inherent nature.

## Competing interests

The authors declare that they have no competing interests. Each author certifies that he has no commercial associations (e.g., consultancies, stock ownership, equity interests, patent/licensing arrangements, etc.) that might pose a conflict of interest in connection with the submitted article.

## Authors' contributions

HNM has contributed in conception, design, acquisition of data, analysis and interpretation of data, drafting the manuscript and revising it critically, and guiding the experiment; BP and JYH contributed in acquisition of data, revising the manuscript critically, and giving the final approval; SWS contributed in conception and design of data, drafting the manuscript, and giving the final approval; JHY, YHP, JML and YHK have contributed in analysis of data and performing the experiment. All authors read and approved the final manuscript.
